# Meningococcal, influenza virus, and hepatitis B virus vaccination coverage level among health care workers in Hajj

**DOI:** 10.1186/1471-2334-7-80

**Published:** 2007-07-18

**Authors:** Tariq A Madani, Tawfik M Ghabrah

**Affiliations:** 1 Department of Medicine, Faculty of Medicine, King Abdulaziz University, Jeddah, Kingdom of Saudi Arabia; 2 The Ministry of Health, Riyadh, Kingdom of Saudi Arabia; 3 Department of Family and Community Medicine, Faculty of Medicine, King Abdulaziz University, Jeddah, Kingdom of Saudi Arabia

## Abstract

**Background:**

The objective of this study was to assess the compliance of health care workers (HCWs) employed in Hajj in receiving the meningococcal, influenza, and hepatitis B vaccines.

**Methods:**

A cross-sectional survey of doctors and nurses working in all Mena and Arafat hospitals and primary health care centers who attended Hajj-medicine training programs immediately before the beginning of Hajj of the lunar Islamic year 1423 (2003) using self-administered structured questionnaire which included demographic data and data on vaccination history.

**Results:**

A total of 392 HCWs were studied including 215 (54.8%) nurses and 177 (45.2%) doctors. One hundred and sixty four (41.8%) HCWs were from Makkah and the rest were recruited from other regions in Saudi Arabia. Three hundred and twenty three (82.4%) HCWs received the quadrivalent (ACYW135) meningococcal meningitis vaccine with 271 (83.9%) HCWs receiving it at least 2 weeks before coming to Hajj, whereas the remaining 52 (16.1%) HCWs received it within < 2 weeks. Only 23 (5.9%) HCWs received the current year's influenza virus vaccine. Two hundred and sixty (66.3%) of HCWs received the three-dose hepatitis B vaccine series, 19.3% received one or two doses, and 14.3% did not receive any dose. There was no statistically significant difference in compliance with the three vaccines between doctors and nurses.

**Conclusion:**

The meningococcal and hepatitis B vaccination coverage level among HCWs in Hajj was suboptimal and the influenza vaccination level was notably low. Strategies to improve vaccination coverage among HCWs should be adopted by all health care facilities in Saudi Arabia.

## Background

Ensuring that health care workers (HCWs) are immune to vaccine-preventable diseases is an essential part of successful employee health programs. Optimal use of vaccines can prevent transmission of vaccine-preventable diseases and eliminate unnecessary work restriction. Prevention of illness through comprehensive HCWs immunization programs is far more cost-effective than case management and outbreak control [1]. In Saudi Arabia, the national vaccination recommendations for HCWs are generally the same as those recommended by the Centers for Disease Control and Prevention [2]. Meningococcal vaccination of all HCWs is additionally recommended in Saudi Arabia, particularly for those working directly with pilgrims in Hajj. Influenza and hepatitis B vaccines are also emphasized for this group of HCWs. These three and other required vaccines are made available free of charge for all HCWs in all health care facilities in Saudi Arabia. The objective of this study was to assess the compliance of HCWs employed in Hajj in receiving the meningococcal, influenza, and hepatitis B vaccines that are particularly recommended for Hajj by the Ministry of Health.

## Methods

### Hajj

Hajj is the fifth of the five pillars of Islam. Any healthy Muslim adult is required to perform Hajj once in his/her life if he/she is financially and physically capable. The Hajj begins on the 8^th ^day of Dhul-Hijjah, the 12^th ^month of the lunar Islamic year, and ends on the 13^th ^day of the same month. Hajj has to be performed in three main locations in Makkah, namely, the sacred Kaaba (in the holy city of Makkah), and Mena and Arafat, which are approximately 5 and 18 Kilometers far from Makkah, respectively. Approximately, 2–3 million pilgrims perform Hajj every year; one third of whom come from within Saudi Arabia and two thirds come from other countries. Most pilgrims stay in fire-resistant air-conditioned camping tents in Mena during the entire Hajj period. Financially deprived pilgrims who cannot afford to pay for the cost of staying in camps usually stay outdoor. Free medical care services are provided to pilgrims by the Saudi Ministry of Health.

### Study design and population

A cross-sectional survey was conducted in Mena and Arafat during the Hajj season of the Islamic year 1423 corresponding to 2003 Gregorian. HCWs included in the study were all doctors and nurses working in Mena and Arafat hospitals and primary health care centers who attended Hajj-medicine training programs immediately before the beginning of Hajj. Many of the HCWs were recruited from various regions in Saudi Arabia to supplement the local HCWs working in Makkah to cover the extensive medical services provided to pilgrims in Hajj.

### Data collection

The Saudi Ministry of Health approved the study. Data were collected using a structured questionnaire which included demographic data and data on vaccination history. The questionnaire was anonymously self-administered by HCWs with supervision from the research team members.

### Data analysis

Data entry and statistical analysis were performed using computer and the Statistical Package for Social Science (SPSS) program. Descriptive statistics, cross tabulations, and Chi-square test were performed as appropriate. Multiple logistic regression analysis was performed to identify predictors of compliance to vaccination after controlling for possible confounding factors. Statistical significance was set at < 0.05.

## Results

A total of 392 HCWs attended the Hajj-medicine training programs. All HCWs agreed to participate in the study with 100% response rate. Two hundred and fifteen (54.8%) HCWs were nurses and 177 (45.2%) HCWs were doctors. Three hundred and fifteen (80.4%) HCWs worked in hospitals, whereas 77 (19.6%) HCWs worked in primary health care centers during this Hajj season. Of the 392 HCWs, 164 (41.8%) HCWs were from Makkah, 85 (21.8%), from Riyadh, 53 (13.6%), from the Eastern Region, 28 (7.3%), from Asir, 21 (5.3%), from Al-Qassim, 13 (3.4%), from Najran, 8 (1.9%), each from Al-Baha and Jizan, and 4 (1.0%), each from Al-Jouf, Hail, and Northern Region.

Table 1 shows the socio-demographic characteristics of HCWs included in the study. Of the 392 HCWs, 323 (82.4%) HCWs received the quadrivalent (ACYW135) meningococcal vaccine recommended by the Ministry of Health. There was no statistically significant difference in compliance with this vaccine between doctors and nurses (78.9% versus 85.4%, respectively, p = 0.172). Of the 323 (82.4%) HCWs who received the meningococcal vaccine, 271 (83.9%) HCWs received it at least 2 weeks and not more than 3 years before coming to Hajj as recommended by the Ministry of Health, whereas the remaining 52 (16.1%) HCWs received it within less than 2 weeks.

**Table 1:** Socio-demographic characteristics of 177 doctors and 215 nurses included in the study

Characteristic	Number	Percent
Sex:		
Doctors:		
Male	142	80.2
Female	35	19.8
Nurses:		
Male	49	22.8
Female	166	77.2
Age group (year)		
Doctors		
20–30	7	4.0
31–40	71	40.1
41–50	79	44.6
51–60	20	11.3
Nurses		
20–30	100	46.5
31–40	69	32.1
41–50	33	15.3
51–60	13	6.1
Nationality:		
Doctors:		
Saudi	46	26.0
Non-Saudi	131	74.0
Nurses:		
Saudi	62	28.8
Non-Saudi	153	71.2
Years of experience		
Doctors		
1–10	68	38.4
11–20	85	48.0
21 or more	24	13.6
Nurses		
1–10	115	53.5
11–20	78	36.3
21 or more	22	10.2
Degree:		
Doctors:		
Bachelor	52	29.4
Master/Diploma	72	40.7
Doctorate/Board	53	29.9
Nurses:		
Diploma	137	63.7
Bachelor	78	36.3
Position		
Doctors:		
General Practitioner	30	16.9
Resident	57	32.2
Specialist	69	39.0
Consultant	21	11.9
Nurses		
Staff nurse	192	89.3
Head nurse	23	10.7
Original work place		
Makkah	164	41.8
Riyadh	85	21.8
North region	4	1.0
Eastern region	53	13.6
Jazan	8	1.9
Asir	28	7.3
Najran	13	3.4
Baha	8	1.9
Qaseem	21	5.3
Aljouf	4	1.0
Hail	4	1.0

Only 23 (5.9%) HCWs received the current year's influenza virus vaccine. There was no statistically significant difference in compliance with this vaccine between doctors and nurses (5.6 % versus 7.4 %, respectively, p = 0.573).

Of the 392 HCWs, 260 (66.3%) HCWs completed the three-dose series of the hepatitis B vaccine, 48 (12.2%) HCWs received 2 doses, 28 (7.1%) HCWs received one dose, and 56 (14.3 %) HCWs did not receive any dose of the vaccine. There was no statistically significant difference in compliance with hepatitis B vaccination between doctors and nurses (Table 2). Of the 260 HCWs who completed the hepatitis B vaccination series, 134 (51.5%) HCWs had post-vaccination tests to confirm development of immunity.

**Table 2:** Compliance of 392 health care workers (177 doctors and 215 nurses) with the hepatitis B virus vaccination

Number of doses	Overall %	% of doctors	% of nurses
None	14.3	14.4	14.1
1 dose	7.1	7.2	6.8
2 doses	12.2	16.2	9.2
3 or more doses	66.3	62.3	69.9

X^2^_df = 6 _= 11.25, p-value = 0.081.

Table 3 shows a bivariate analysis of the compliance of 392 health care workers for receiving the quadrivalent meningococcal meningitis, influenza, and hepatitis B vaccines according to their gender, age group, nationality, years of experience, degree, position, and original place of work. This analysis showed no statistically significant difference in compliance with the receipt of the vaccines among the various groups with two exceptions; the compliance of non-Saudi HCWs for the receipt of the meningitis vaccine was significantly higher than that of the Saudi mates (84.2% versus 77.8%, p = 0.027), and the compliance of HCWs with lower degrees (diploma and bachelor) was significantly higher than that of HCWs with higher degrees (p = 0.011). Multivariate logistic regression analysis confirmed that non-Saudi nationality was an independent predictor of a better compliance with the meningitis vaccine (odds ratio = 3.28, 95% confidence interval = 1.17 – 9.21, p = 0.024). However, lower degrees were not independent predictors of a better compliance with this vaccine (p = 0.797).

**Table 3:** Bivariate analysis of the compliance of 392 health care workers (HCWs) for receiving the ACYW135 meningococcal meningitis, influenza, and at least one dose of the hepatitis B vaccines according to their gender, age group, nationality, years of experience, degree, position, and original place of work.

	Number of HCWs	HCWs who received the meningococcal vaccine (%)	P	HCWs who received the influenza vaccine (%)	P	HCWs who received at least one dose of the hepatitis B vaccine (%)	P
Total number of HCWs	392	323 (82.4)		23 (5.9)		336 (85.7)	
Sex							
Male	191	150 (78.5)	0.327	9 (4.7)	0.319	157 (82.2)	0.567
Female	201	173 (86.1)		14 (7.0)		179 (89.1)	
Age group (year)							
20–30	107	92 (86.0)	0.972	5 (4.7)	0.683	95 (88.8)	0.156
31–40	140	113 (80.7)		8 (5.7)		125 (89.3)	
41–50	112	93 (83.0)		6 (5.4)		88 (78.6)	
51–60	33	25 (75.8)		4 (12.1)		28 (84.8)	
Nationality							
Saudi	108	84 (77.8)	0.027	7 (6.5)	0.680	90 (83.3)	0.104
Non-Saudi	284	239 (84.2)		16 (5.6)		246 (86.6)	
Experience in year							
1–10	183	154 (84.2)	0.903	8 (4.4)	0.108	164 (89.6)	0.149
11–20	163	133 (81.6)		14 (8.6)		132 (81.0)	
21 or more	46	36 (78.3)		1 (2.2)		40 (87.0)	
Degree							
Diploma	137	111 (81.0)	0.011	8 (5.8)	0.204	110 (80.3)	0.088
Bachelor	130	116 (89.2)		8 (6.2)		117 (90.0)	
Master/high diploma	72	60 (83.3)		2 (2.8)		60 (83.3)	
Doctorate/Board	53	36 (67.9)		5 (9.4)		49 (92.5)	
Position							
Consultant	21	16 (76.2)	0.500	3 (14.3)	0.351	19 (90.5)	0.810
Specialist	69	54 (78.3)		3 (4.3)		62 (89.9)	
Resident	57	46 (80.7)		1 (1.8)		46 (80.7)	
General practitioner	30	23 (76.7)		2 (6.7)		24 (80.0)	
Head nurse	23	16 (69.6)		2 (8.7)		18 (78.3)	
Nurse	192	168 (87.5)		12 (6.3)		167 (87.0)	
Original work place							
Makkah	164	136 (82.9)	0.574	12 (7.3)	0.651	136 (82.9)	0.643
Riyadh	85	75 (88.2)		2 (2.4)		73 (85.9)	
North region	4	2 (50.0)		0		2 (50.0)	
Eastern region	53	45 (84.9)		4 (7.5)		49 (92.5)	
Jazan	8	6 (75.0)		0		8 (100.0)	
Asir	28	21 (75.0)		1 (3.6)		24 (85.7)	
Najran	13	12 (92.3)		2 (15.4)		13 (100.0)	
Baha	8	8 (100.0)		0		4 (50.0)	
Qaseem	21	14 (66.7)		2 (9.5)		19 (90.5)	
Aljouf	4	2 (50.0)		0		4 (100.0)	
Hail	4	2 (50.0)		0		4 (100.0)	

## Discussion

Until year 2000, bivalent (AC) meningococcal vaccination was recommended for all people coming to the Hajj areas from inside or outside Saudi Arabia. Since 2000, *Neisseria meningitides *W135 caused outbreaks among pilgrims for the first time in history [3]. As a result, since year 2000, the quadrivalent (ACWY135) meningococcal vaccination has been recommended by the Ministry of Health for all people coming for Hajj with revaccination every 3 years if the unconjugated polysaccharide vaccine was used. The quadrivalent meningococcal polysaccharide vaccine has been made available free of charge in all health care facilities for all HCWs as well as for other people within Saudi Arabia intending to perform Hajj. This study showed that compliance of HCWs with this vaccine was suboptimal (82.4%) with almost one fifth of HCWs being unvaccinated. Non-Saudi HCWs were significantly more compliant with this vaccine than Saudi HCWs. Further studies are needed to determine the reasons for the lower compliance among the Saudi HCWs. Possible explanations for this difference include better awareness of the importance of this vaccine among the non-Saudis and false sense of security and perceived low likelihood of contracting meningococcal meningitis among Saudi HCWs. Gender, age, years of experience, qualification degree, position, and original place of work were not significant predictors of compliance to the meningococcal vaccine.

During community influenza outbreaks, admitting patients infected with influenza to hospitals has led to nosocomial transmission of the disease, including transmission from staff to patients [4-6]. Transmission of influenza among medical staff causes absenteeism and considerable disruption of health care [7-10]. The Healthcare Infection Control Practices Advisory Committee (HICPAC) and the Advisory Committee on Immunization Practices (ACIP) in the United States recommend that all HCWs be vaccinated annually against influenza [11]. However, in spite of a long-standing recommendation for influenza vaccination of HCWs, and the availability of a safe and effective vaccine, influenza vaccination coverage levels among HCWs remain substantially low worldwide [11]. For instance, during 1989–2003, HCWs influenza vaccination coverage levels in the United States increased from 10% to 40%; however, coverage levels have remained relatively constant since 1997 [12]. Substantially lower vaccination rates have been reported among HCWs who have contact with certain populations at high risk in the United States [13-16]. Low influenza vaccination coverage rates have also been reported from Europe [17-20]. This vaccine is particularly recommended for HCWs working in Hajj because of the very high risk of acquiring influenza infection in such overcrowded situations. However, in this study, only 5.9% of HCWs received the current year's influenza virus vaccine. Gender, age, nationality, years of experience, qualification degree, position, and original place of work were not significant predictors of compliance to this vaccine. Reported barriers to HCWs receipt or acceptance of influenza vaccination include fear of vaccine side effects, insufficient time or inconvenience, perceived ineffectiveness of the vaccine, medical contraindication, perceived low likelihood of contracting influenza, reliance on treatment with homeopathic medications, avoidance of medications, and fear of needles [11].

Hepatitis B virus (HBV) infection is a major infectious hazard for HCWs [11,12]. In general, the seroprevalence of HBV among HCWs is twofold to fourfold higher than that of blood donor controls [23]. The risk for acquiring HBV infection from occupational exposures is dependent on the frequency of percutaneous and permucosal exposures to blood or body fluids containing blood [22]. The risk for transmission from a single needlestick varies according to hepatitis B e antigen status: 1% to 6% for e antigen-negative blood compared with 22% to 40% with e antigen-positive blood [23]. Not all cases of HBV transmission are explained by needlesticks, suggesting that other modes of spread may be possible [23]. HBV vaccination of all HCWs has been strongly recommended [2]. The HBV three-dose vaccine series is 88% effective [24,25]. From 1985 to 1994, the use of HBV vaccine and adherence to other preventive measures such as standard precautions lead to a 90% decline of HBV infection among HCWs in the United States [26,27]. Even though, HBV vaccination of all HCWs has been strongly recommended, the vaccination coverage level of HCWs remains suboptimal with many studies reporting rates between 40–86% [28-35]. A recent study from Saudi Arabia reported an overall compliance rate of 71.6% (932/1302) among HCWs [36]. In that study the compliance rate was 79.5% (492/619) among nurses, 78.3% (242/309) among technicians, and 52.9% (198/374) among physicians [36]. In the current study, the compliance rate was similar to earlier reports as only 66.3% of HCWs received the three-dose vaccine series, 19.3% received one or two doses, and 14.3% did not receive any dose. Gender, age, nationality, years of experience, qualification degree, position, and original place of work were not significant predictors of compliance to this vaccine.

Several strategies for improving HCWs vaccination coverage have been recommended [11]. Successful HCWs vaccination programs are multifaceted and combine publicity and education to combat fears and misconceptions about vaccines, use of reminder recall systems, efforts to remove administrative and financial barriers, role modeling, and monitoring and feedback on vaccination coverage [11]. Educational programs should emphasize the benefits of HCWs vaccination for staff and patients [11]. Organized campaigns that promote and make vaccine accessible can improve vaccination rates among HCWs [11]. Vaccination of senior medical staff or opinion leaders has been associated with higher vaccination acceptance among staff members under their leadership [11]. Removing administrative barriers such as costs and providing vaccine in locations and at times easily accessible by HCWs can substantially improve vaccine acceptance[11]. Making vaccine readily accessible at congregate areas (e.g., clinics), during conferences, or by use of mobile carts has been demonstrated to improve vaccination coverage rates [11]. Monitoring vaccination coverage by facility area (e.g., ward or unit) or occupational group allows facilities to identify where vaccination levels are low and interventions that should be targeted. Obtaining declination statements from HCWs who refuse vaccination for reasons other than medical contraindications can assist facilities in identifying personnel who might require targeted education or other interventions to overcome barriers to vaccine acceptance. In addition, collection of such information will allow health-care facilities to determine what proportion of their staff are reached and offered vaccine [11].

## Conclusion

The meningococcal and hepatitis B vaccination coverage level among HCWs in Hajj was suboptimal and the influenza vaccination rate was notably low. Recommended strategies to improve vaccination coverage among HCWs should be adopted by all health care facilities in Saudi Arabia to ensure adequate vaccination coverage particularly with the Hajj-related vaccines.

## Competing interests

The author(s) declare that they have no competing interests.

## Authors' contributions

TAM designed the study and wrote the manuscript. TMG participated in the design of the study, performed the statistical analysis, and read and approved the manuscript.

## Pre-publication history

The pre-publication history for this paper can be accessed here:


